# Functional Connectivity Disruption in Neonates with Prenatal Marijuana Exposure

**DOI:** 10.3389/fnhum.2015.00601

**Published:** 2015-11-04

**Authors:** Karen Grewen, Andrew P. Salzwedel, Wei Gao

**Affiliations:** ^1^Department of Psychiatry, Neurobiology, and Psychology, University of North Carolina Chapel Hill, Chapel Hill, NC, USA; ^2^Department of Radiology, Biomedical Research Imaging Center, University of North Carolina Chapel Hill, Chapel Hill, NC, USA; ^3^Department of Biomedical Sciences and Imaging, Biomedical Imaging Research Institute, Cedars-Sinai Medical Cente, Los Angeles, CA, USA

**Keywords:** caudate, cerebellum, functional connectivity, insula, neonatal, prenatal marijuana, resting state

## Abstract

Prenatal marijuana exposure (PME) is linked to neurobehavioral and cognitive impairments; however, findings in childhood and adolescence are inconsistent. Type-1 cannabinoid receptors (CB1R) modulate fetal neurodevelopment, mediating PME effects on growth of functional circuitry sub-serving behaviors critical for academic and social success. The purpose of this study was to investigate the effects of prenatal marijuana on development of early brain functional circuitry prior to prolonged postnatal environmental influences. We measured resting state functional connectivity during unsedated sleep in infants at 2–6 weeks (+MJ: 20 with PME in combination with nicotine, alcohol, opiates, and/or selective serotonin reuptake inhibitors; −MJ: 23 exposed to the same other drugs without marijuana, CTR: 20 drug-free controls). Connectivity of subcortical seed regions with high fetal CB1R expression was examined. Marijuana-specific differences were observed in insula and three striatal connections: anterior insula–cerebellum, right caudate–cerebellum, right caudate–right fusiform gyrus/inferior occipital, left caudate–cerebellum. +MJ neonates had hypo-connectivity in all clusters compared with −MJ and CTR groups. Altered striatal connectivity to areas involved in visual spatial and motor learning, attention, and in fine-tuning of motor outputs involved in movement and language production may contribute to neurobehavioral deficits reported in this at-risk group. Disrupted anterior insula connectivity may contribute to altered integration of interoceptive signals with salience estimates, motivation, decision-making, and later drug use. Compared with CTRs, both +MJ and −MJ groups demonstrated hyper-connectivity of left amygdala seed with orbital frontal cortex and hypo-connectivity of posterior thalamus seed with hippocampus, suggesting vulnerability to multiple drugs in these circuits.

## Introduction

Marijuana (cannabis) is currently the most frequently used illicit drug during pregnancy. Prenatal prevalence is approximately 5.2%, with significantly higher rates seen in younger pregnant women (Warner et al., 2014). Reports of developmental deficits in infancy, childhood, and adolescence associated with prenatal marijuana exposure (PME) suggest long-lasting effects on brain and behavior but results are inconsistent (Huizink, 2014). However, such behavioral findings may be confounded by postnatal environmental factors (Fried and Smith, 2001) over the often long interval between exposure and behavioral assessment. Moreover, findings from earlier studies may not reflect current levels of toxicity associated with recent increases in consumption, potency, and newer marijuana delivery systems (e.g., e-joints) (Volkow et al., 2014). Nevertheless, some but not all, studies of PME report increased startles and tremors and reduced visual habituation in infancy (Fried, 1995, 1996), impaired short-term memory, verbal reasoning and attention and increased impulsivity and hyperactivity in early childhood, and deficits in abstract and visual reasoning, problem-solving, sustained attention (Fried, 2002), visual-motor coordination (Willford et al., 2010), and increased risk for illicit drug use (Spano et al., 2010; Calvigioni et al., 2014) in adolescence.

Prenatal marijuana exposure may contribute to such behavioral effects by altering activity within the endocannabinoid (eC) system during *in utero* brain development. The primary psychoactive compound in marijuana, Δ^9^-tetrahydrocannabinol (THC), is an exogenous cannabinoid which crosses the placenta (Behnke and Eyler, 1993) and blood–brain barrier (Schou et al., 1977) to bind to type-1 cannabinoid receptors (CB1R). eC signaling plays a critical role in control of neurogenesis and phenotypic specification of immature neurons (Harkany et al., 2008), and in establishment of the normal fetal neuronal network architecture [e.g., enabling projection axons to reach their specific targets, modulating growth cone structure, motility, and directionality (Gaffuri et al., 2012)]. CB1R is present in both dendrites and growth cones of developing neurons, and additionally shapes network connections by regulating neurite growth and synaptogenesis (Vitalis et al., 2008). In addition, THC binding to CB1R during gestation alters development of central dopamine (DA) and opioid neurotransmitter systems in brain areas regulating reward and motivation, which may increase vulnerability to future drug use and addiction in later life (Spano et al., 2007). Postmortem examination of human fetal brains with PME show disruption of components of developing DA and opioid systems in striatal (DA D2 receptor, opioid precursor genes) and mesocorticolimibic (DA D2 receptor, mu and kappa opioid receptor expression) regions (Wang et al., 2006). Some or all of these essential processes may be reactive to exogenous cannabinoids such as THC during gestation, which is a critical period when brain structure and connectivity undergo massive growth and organization (Knowles, 2012).

Human brain imaging studies show that functional network activity underlies the typical cognitive and behavioral processes reportedly altered by PME, and that aberrant connectivity is linked to atypical functional development in other disorders (Bressler and Menon, 2010; Insel, 2010). A small number of research groups have begun to document how and when these functional networks develop in typical neonates (Lin et al., 2008; Gao et al., 2009; Fransson et al., 2013), and have shown that prenatal exposure to other psychoactive drugs alters early structure (Grewen et al., 2014; Knickmeyer et al., 2014) and connectivity (Salzwedel et al., 2015). Animal studies show prenatal THC-induced disruption of neural connectivity that results in long-lasting alterations in structure and function of cortical circuitry (Tortoriello et al., 2014). However, very little is known about the effects of PME on early brain development in human infants, or on the formation of early functional networks that may underlie the cognitive and behavioral deficits reported in studies of exposed children.

The purpose of the current study was to examine the effects of PEM on functional connectivity in human infants at a time proximal to *in utero* exposure, in order to limit the influence of postnatal environmental differences. We used resting state functional connectivity methods to compare 2- to 6-week-old infants with or without PME. Because a majority of mothers who use marijuana during pregnancy use other psychoactive drugs as well, we compared infants with PME in combination with alcohol, nicotine, opiates, and/or selective serotonin reuptake inhibitors (SSRI) (+MJ) to infants exposed to these same drugs but without marijuana (−MJ). A second control group consisted of drug-naïve control infants (CTR). The hippocampus, insula, amygdala, caudate, putamen, and thalamus were selected as seed regions, given their high levels of CB1R expression in both adult and fetal and neonatal brain (Glass et al., 1977), the critical significance of these structures in early brain functional development [insula (Alcauter et al., 2015), thalamus (Alcauter et al., 2014)], and the reported disruptions of connectivity related to prenatal exposure to other psychoactive drugs (Salzwedel et al., 2015). We expected both marijuana-specific and drug-common functional connectivity alterations in neonates with corresponding drug exposures, based on the significant impact of PME (Filbey and DeWitt, 2012) and other drugs (Kravitz et al., 2015) on establishment of fetal neural circuitry.

## Materials and Methods

### Participants

Infants were participants in a study of the neurodevelopmental effects of prenatal cocaine and other drug exposures (Salzwedel et al., 2015). The results reported here are based on a subset (*N* = 63) of the full cohort which was specifically selected to explore the effects of PEM. All were from the non-cocaine-exposed comparison groups. Infants (29 males and 34 females) were categorized into one of three groups: 20 marijuana positive (+MJ) with or without *in utero* exposure to alcohol, nicotine, SSRI, and opiates (i.e., heroin, oxycontin, methadone, and/or the mixed agonist/antagonist, suboxone); 23 infants with *in utero* exposure to some combination of the aforementioned drugs −MJ; 20 age-matched drug-free controls (CTR). Infants were medically healthy singletons, born at ≥36 weeks gestation. Pregnant women were recruited in the third trimester of pregnancy. Primary recruitment sites for drug-exposed mother–infant dyads were local residential and outpatient treatment programs for women with perinatal substance abuse and their children. In addition, both CTR and drug-exposed mothers were recruited from local obstetric clinics for low income women, and from local advertisements and Craigslist.

#### Drug-Exposure Status

Prenatal drug exposure was assessed by maternal Time Line Follow Back (TLFB) interview (Sobell and Sobell, 1995) and confirmed by perinatal medical record review of prenatal urine toxicology or infant meconium at delivery (available for ~65% of participants). The TLFB is a psychometrically sound instrument for assessing alcohol/drug use, with test–retest reliability over periods from 1 to 12 months in varied populations (Vakili et al., 2008; Robinson et al., 2014). The TLFB interview for detection of illicit cocaine, cannabis, and opiates appears to give highly accurate estimates of substance use in both clinical trials and prospective studies when confirmed by biological tests (urine, hair) (Hjorthoj et al., 2012). Meta-analyses reveal lowest and highest weighted averages for accuracy of cannabis, 87.3% (95% confidence interval 86.9–87.7%) and 90.9% (90.5–91.4%); for cocaine, 79.3% (79.1–79.6%) and 84.1% (83.9–84.2%); for opiates 94.0% (93.5–94.5%) (Hjorthoj et al., 2012). Similarly, TLFB reports of alcohol use have been confirmed as highly valid by transdermal testing (Simons et al., 2015). We also used strategies shown to enhance accuracy of self-reports, including assuring participants of confidentiality and informing them of the NIH Certificate of Confidentiality obtained by the study, use of clinically trained interviewer (licensed clinical social worker) and validation with biological measures (prenatal urine or meconium toxicology reports gleaned from medical records for all groups).

Sample characteristics evaluated for incorporation into subsequent analyses included gestational age at birth (days), postnatal age (gestational age at time of MRI scan in days since conception), birth weight, categorical drug exposure (Yes or No to Nicotine, Alcohol, SSRI, Opiates). Socioeconomic status, indexed by maternal education, and maternal depressed affect, indexed by the Edinburgh Postnatal Depression Scale (Murray and Carothers, 1990), were assessed at infant MRI visit. Maternal education was determined by self-report, and ranged from some high school to post-graduate work. Rank scores were coded as: some high school = 3, graduated from high school = 4, trade school or business college = 5, some college = 6, graduated with 4-year college degree = 7, and post-graduate work at university = 8. Maternal education data was missing for 15 mothers, and maternal depression values were missing for 4 mothers. Group means were compared statistically using analyses of variance (ANOVA); group proportions were tested using the chi-square statistic (X^2^). Independent two-tailed *t*-tests were used to compare differences in drug use frequency between +MJ and −MJ groups, with Satterwaite *p*-values reported when group values had unequal variances (Table S1 in Supplementary Material). This study was approved by the Biomedical Institutional Review Board of the University of North Carolina and all mothers granted their written informed consent for themselves and their infants.

### MR Image Acquisition

Infants were scanned during sleep without sedation at 2–6 weeks after birth. Infants were first fed and swaddled. When asleep each was fitted with ear protection and his/her head was secured in a vacuum-fixation device within the scanner. Each was monitored by sight, touch, and by pulse oximetry for heart rate and percentage of oxygen saturation throughout the scan. Data were collected using two scanners: scanner 1 = 3 T head only Siemens Allegra with circular polarization head coil (*n* = 47), Scanner 2 = 3 T Siemens Tim Trio with 32-channel head coil (*n* = 16) (Siemens Medical Solutions). The number of subjects per group per scanner varied (Scanner 1: 15 +MJ, 19 −MJ, 13 CTR; Scanner 2: 5 +MJ, 4 −MJ, 7 CTR). Scanner assignment was included in statistical analyses. T1-weighted structural images were collected using a 3D magnetization prepared rapid gradient echo (MP-RAGE) pulse sequence: repetition time (TR) = 1820 ms, echo time (TE) = 3.75 ms, inversion time (TI) = 1100 ms, flip angle = 7°, 144 slices, voxel size = 1 mm^3^. Resting state functional magnetic images (rsfMRI) were acquired using a T2*-weighted echo planar imaging (EPI) pulse sequence: TR = 2s, TE = 32 ms, 33 slices, voxel size = 4 mm^3^, number of volumes = 150 (5-min duration).

### Image Preprocessing

Functional data were preprocessed using the Functional MRI of the Brain (FMRIB) Software Library (FSL; version 4.1.9) (Jenkinson et al., 2012). Steps included discarding the first 10 volumes (20 s), slice-timing correction, rigid-body motion correction, spatial smoothing (Gaussian kernel FWHM of 6 mm), bandpass filtering (0.01–0.08 Hz), and regression of whole brain [global-signal regression (GSR)], white matter, CSF, and the six motion parameters. Data scrubbing was also implemented to reduce the effect of motion in rsfMRI analyses; scrubbing criteria: 0.5% signal change and 0.5 mm frame-wise displacement. The number of volumes removed and residual frame-wise displacement were compared using individual ANOVAs to ensure motion was consistent between groups. Number of volumes removed from each of the three groups was statistically indistinguishable: *F*_Volumes_(2,62) = 0.28, *p* = 0.76, +MJ 7.95 ± 3.30 (SEM), −MJ 8.09 ± 2.59, CTR 5.50 ± 2.18; Residual frame-wise displacement; *F*_FD_(2,62) = 0.63, *p* = 0.54, +MJ 0.15 ± 0.03, −MJ 0.12 ± 0.01, CTR 0.13 ± 0.02. FSL and the Analysis of Functional NeuroImages software suite [AFNI version 2011-12-21-1014; (Cox, 1996)] were used to process the structural images. Structural image skull stripping was done using a two-step process. First, FSL-bet2 was used to perform an initial skull strip, and then the result was bolstered using the AFNI script @NoisySkullStrip.

Alignment of functional data into a common space involved two steps: (1) within-subject rigid alignment [FSL FLIRT (for FMRIB Linear Image Restoration Tool)] between functional and T1-weighted images; (2) non-linear [FSL FNIRT (for FMRIB Non-linear Image Registration Tool)] registration of the T1-weighted images to a T1-weighted template image acquired from an independent subject scanned at 2 weeks of age. The combined transformation field (linear plus non-linear) was used to warp the preprocessed rsfMRI data to the template space. Alignment was inspected visually for quality across all subjects.

### Functional Connectivity Analyses

Standard seed-based whole-brain functional connectivity analyses were carried out using the temporal correlation method (Biswal et al., 1995). The amygdala, hippocampus, putamen, anterior/posterior insula, caudate, and anterior/posterior thalamus were used as seed regions in the analyses. Except for the thalamus and insula, all other seed regions were defined using the Harvard–Oxford probabilistic atlas provided with FSL (Desikan et al., 2006). The atlas was warped into the study-specific template space using 4D HAMMER (Shen and Davatzikos, 2004). Masks from one of our previous functional parcelation studies of the insula (Alcauter et al., 2015) were used to define an anterior and a posterior insula seed given the reported functional segregation. Similarly, an anterior and a posterior thalamus seeds were defined due to the observed biases in their functional connectivity to frontal and sensorimotor regions of the brain, respectively. Specifically, we determined the center of mass of the previously defined anterior and posterior thalamic cluster with specific cortical connections and defined the seed regions as the center voxel plus the face-connected neighboring voxels. The posterior thalamus definition included two seeds since the original posterior thalamus parcelation contained two largely hemi-symmetrical clusters. Next, the average time series of each seed was extracted and used to perform whole-brain correlation analyses. The correlation measures were Fisher-z transformed and compared within and between groups (Chen et al., 2014). Within groups, one-sample *t*-tests were applied to generate group-specific functional connectivity maps. Between groups, multivariate analysis of variance modeling was used to detect voxel-wise differences in functional connectivity at the whole-brain level (i.e., previous functional connectivity maps were not used to mask the data) while controlling for other explanatory variables: gender, scanner, gestational age, birth weight, and postnatal age at scan (mean-centered continuous variables). Significance was determined using a combined approach (Forman et al., 1995), which imposes a minimum *p*-value (i.e., *p* < 0.01) and cluster size (32 voxels) threshold to correct for multiple comparisons (α < 0.05) at the whole-brain level. Between-group differences included the contributions of both positive connectivity and negative connectivity. For the descriptions of results, hyper- and hypo-connectivity are used to represent positive or negative shifts in connectivity relative to CTR, respectively. However, we also use the term “disrupted connectivity” to describe both types of deviations from normal.

For each identified cluster, the mean *Z*-scores were extracted and additional group-wise comparisons were carried out using the ANOVA method (MATLAB–anovan). First, the model used in the 3dMVM analysis was repeated for the cluster-wise averages. Then, the model was expanded to include maternal education and depression levels (*n* = 46), in order to test the effects of all potential explanatory variables. Here, continuous variables were not mean-centered in order to explicitly model the contributions of the combined within- and between-group differences to the observed variations in functional connectivity. Significant ANOVA main effects (*p* ≤ 0.05, Dunn–Sidak corrected for number of seed regions *N* = 14) were followed up with *post hoc* comparisons (MATLAB–multcompare, Dunn–Sidak corrected) on the population marginal means in order to identify significant (*p* ≤ 0.05) pair-wise differences. In order to shed light on the potential effects of GSR in this study and to gain a better understanding of relative group differences, two additional analyses were implemented. Specifically, the cluster-level analyses were repeated using data with, (1) head-motion regression only (i.e., no CSF, WM, or GSR regression), and (2) head-motion regression +*post hoc* standardization (mean subtraction; see Yan et al., 2013).

### Drug Specificity and Interactions

For each detected cluster showing group differences, additional *post hoc* ANOVAs were performed to test the specificity of marijuana effects within the drug-exposed sample. Specifically, we constructed a model with categorical drug exposures (marijuana, nicotine, alcohol, SSRI, opiates) as the main effects, thus, allowing us to test the potential effects of all drugs on the detected functional connectivity alterations for each cluster.

## Results

### Participant Characteristics

Summary statistics for each group are presented in Table 1. Gender, gestational age at birth, postnatal age at MRI, birth weight, and maternal depression levels were similar for all three groups. Maternal education differed significantly between groups, with +MJ < −MJ < CTR. The distribution of categorical non-marijuana drug use did not differ between +MJ and −MJ groups. A large proportion of +MJ and −MJ groups reported prenatal cigarette-smoking (85, 87% respectively) and alcohol use (50, 30%, respectively) in at least one trimester of pregnancy, with similarly smaller rates of prenatal SSRI and opiate use. Table S1 in Supplementary Material displays the number of users and the frequency of use of each drug in +MJ and −MJ groups by trimester and in the postnatal period prior to MRI. No significant differences were observed between +MJ and −MJ groups for cigarettes smoked per day or alcoholic drinks consumed per week in any trimester or in the postnatal period prior to MRI, and both groups appeared to have fewer users and less drug use as pregnancy progressed. The small number of opiate users and heterogeneity of use in each group prevented valid statistical comparison; however, opiate use was more prevalent in the −MJ group (+MJ = 1, −MJ = 5).

**Table 1:** **Participant characteristics**.

	+MJ			−MJ			CTR			*p*
	Mean		SE	Mean		SE	Mean		SE	
Gestational age (days)	280	±	1	279	±	2	280	±	1	0.94
Postnatal age at MRI (days)	304	±	2	305	±	2	306	±	2	0.89
Birth weight (pounds)	7.51	±	0.27	7.51	±	0.24	7.9	±	0.26	0.50
Maternal depressed mood^a^	4.74	±	1.2	5.83	±	1.51	3.4	±	0.78	0.37
Group *n* (total, male, female)	20	12	8	23	7	16	20	10	10	0.14
**Other drug exposures**	***n***	**%**		***n***	**%**				**χ^2^**	***p***
Nicotine	17	0.85		20	0.87				0.034	0.85
Alcohol	10	0.50		7	0.30				1.713	0.19
SSRI	2	0.10		3	0.13				0.096	0.76
Opiates	1	0.05		5	0.22				2.497	0.11

*^a^Total score on Edinburgh Postnatal Depression Scale at MRI visit*.

### Whole-Brain Functional Connectivity

**Figure 1:**
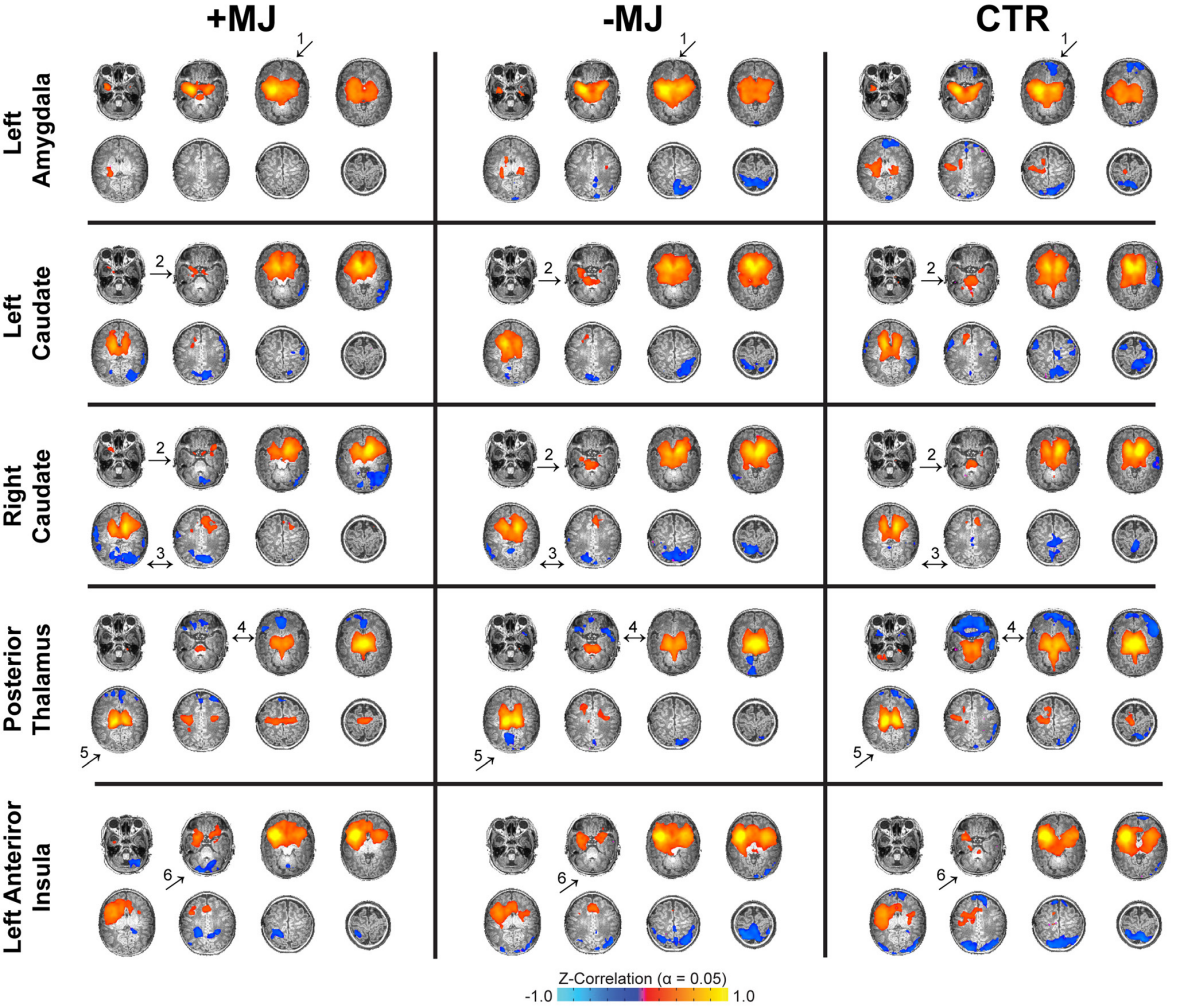
**Neonatal functional connectivity and prenatal marijuana exposure**. Functional connectivity for different seed regions (top to bottom) and infant groups (left to right): marijuana positive (+MJ) with or without *in utero* exposure to alcohol, nicotine, SSRIs, and opiates; infants with *in utero* exposure to some combination of the aforesaid drugs minus marijuana (−MJ); and age-matched drug-free controls (CTR). Data threshold set using the combined approach: α = 0.05: voxel-wise *p* ≤ 0.01, cluster size = 32 voxels. Pseudo-coloring (see color bar at bottom) is based on Fisher’s *Z*-transformation of the temporal correlation between each voxel and the seed region. Data visualized in the axial view on a subset of high-resolution T1-weighted reference images.

**Figure 2:**
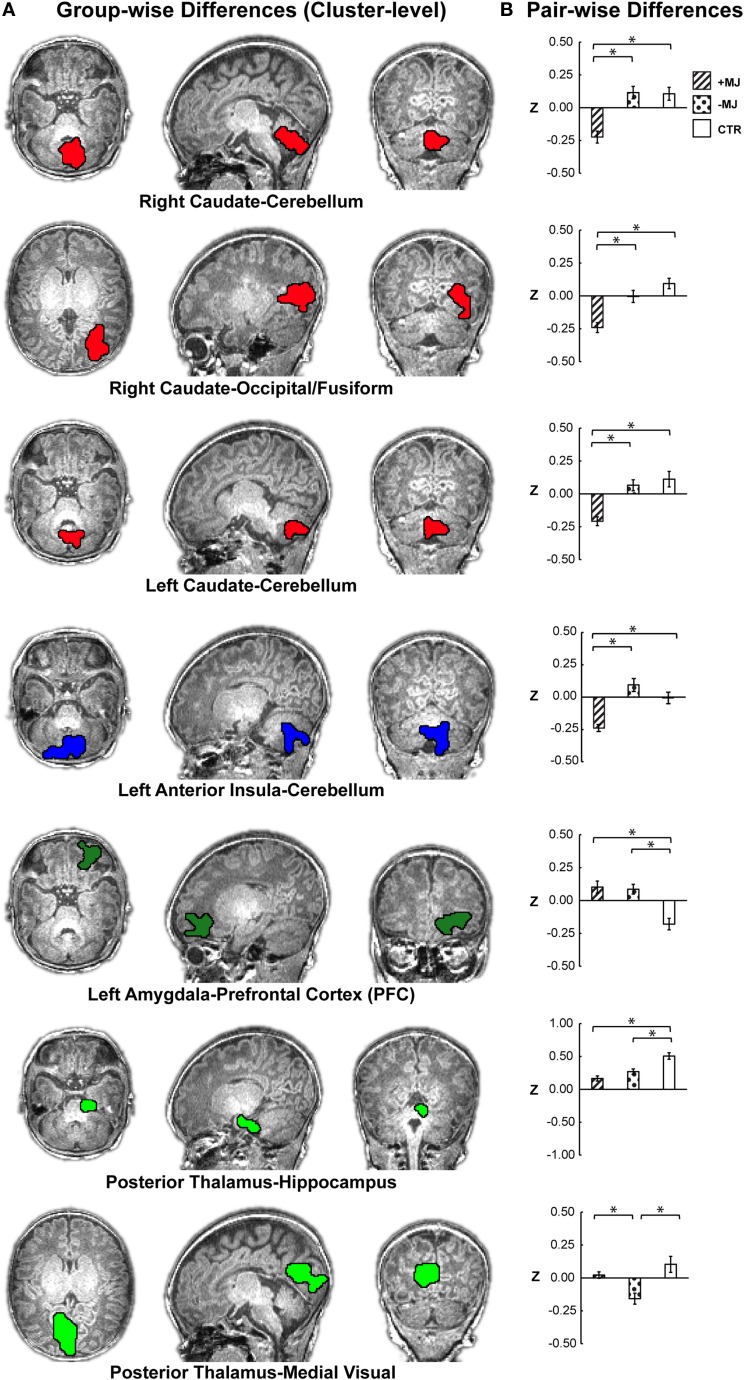
**Localization of group-level (**+**MJ, **−**MJ, CTR) functional connectivity differences at the cluster level and comparisons by group**. **(A)** Clusters depicted on the high-resolution anatomical reference images. Clusters were detected using the combined threshold approach controlling for gestational age, birth weight, scan age, gender, and scanner (α < 0.05): multivariate group-wise difference (+MJ, −MJ, CTR) *p* ≤ 0.01, minimum number of voxels = 32, nearest neighbor clustering (NN) = 3. **(B)** Comparisons of functional connectivity within cluster by neonatal group. (*) indicates significant (*p* ≤ 0.05 Dunn–Sidak corrected) pair-wise differences between groups while accounting for participant characteristics. Data plotted as mean ± SEM.

Whole-brain functional connectivity maps (threshold α = 0.05; voxel-wise *p* ≤ 0.01 + cluster size = 32 voxels) generated using the amygdala, caudate, thalamus, hippocampus, putamen, and insula as seed regions (see Figure S1 in Supplementary Material for seed locations) were visualized on high-resolution anatomical reference images and qualitatively compared across groups (Figure 1). Note, connectivity analyses were conducted using independent left and right hemisphere seeds for all regions except for the thalamus and insula that were segregated according to anterior and posterior sub-divisions (see Figure S2 in Supplementary Material for maps not depicted in Figure 1). In general, connectivity associated with the left and right hemispheres for each region showed a high degree of similarity in terms of overall pattern and laterality. Qualitatively, there were a number of potential differences in functional connectivity patterns between groups that ultimately showed good correspondence at the cluster level (Figure 2). For instance, in both drug-exposed groups (± MJ) negative connectivity between the amygdala and orbital frontal cortex was distinctly lacking compared to the CTR group (Figure 1 ↑_1_). +MJ infants also showed a distinct lack of positive connectivity between caudate and cerebellum/cerebellar vermis regions for both left and right seed regions (Figure 1 ↑_2_). Furthermore, negative connectivity between the caudate and occipital-fusiform areas appeared enhanced in the +MJ group (Figure 1 ↑_3_), and to a lesser degree in −MJ infants. Positive connectivity associated with the posterior thalamus appeared reduced in the drug-exposed groups (Figure 1 ↑_4_), particularly in more centrally located subcortical structures in and near the hippocampus. −MJ infants showed stronger negative connectivity between the posterior thalamus and occipital-lingual cortices (Figure 1 ↑_5_). Anterior insula–cerebellum connectivity was hypo-connective in the +MJ group (Figure 1 ↑_6_). Overall, +MJ neonates showed less bilateral positive connectivity for many of the seed regions tested (also see Figure S1 in Supplementary Material).

### Significant Clusters with Group Differences

Based on voxel-wise ANOVA analysis, seven clusters were detected with significant between-group differences (Figure 2A α = 0.05: group-wise *p* ≤ 0.01, cluster size ≥32 voxels) after controlling for gestational age at birth, postnatal age at MRI, birth weight (continuous covariates mean-centered within-groups), gender, and scanner. The left anterior insula and right/left caudate seed regions yielded group-wise clusters that primarily localized to the cerebellum/cerebellar vermis. The right caudate also produced a cluster in the occipital-fusiform cortex. Group-wise differences for the left amygdala materialized as a large cluster in the orbital frontal or prefrontal cortices. The posterior thalamus seed yielded two clusters, one centrally located encompassing the hippocampus and another in the occipital-lingual cortices. Table 2 lists each cluster and the degree of overlap (>10%) with different regions using the AAL nomenclature. No significant clusters were detected for the anterior thalamus, left/right posterior insula, right anterior insula, or putamen seeds.

**Table 2:** **Cluster AAL Overlap**.

		(%)
**Right caudate–fusiform/occipital (70 voxels)**		
O3D	Occipital_Inf_R	0.25
FUSID	Fusiform_R	0.25
V1D	Calcarine_R	0.12
LINGD	Lingual_R	0.10
O2D	Occipital_Mid_R	0.10
**Right caudate–cerebellum (46 voxels)**		
CERCRU2G	Cerebellum_Crus2_L	0.20
CERCRU2D	Cerebellum_Crus2_R	0.15
VER8	Vermis_8	0.15
CER8D	Cerebellum_8_R	0.13
CER8G	Cerebellum_8_L	0.10
CER9D	Cerebellum_9_R	0.10
**Left caudate–cerebellum (35 voxels)**		
CER8D	Cerebellum_8_R	0.25
CERCRU2G	Cerebellum_Crus2_L	0.16
CERCRU2D	Cerebellum_Crus2_R	0.13
CER8G	Cerebellum_8_L	0.13
VER4_5	Vermis_4_5	0.13
**Left anterior insula–cerebellum (47 voxels)**		
CERCRU2G	Cerebellum_Crus2_L	0.40
CER8D	Cerebellum_8_R	0.23
CERCRU2D	Cerebellum_Crus2_R	0.13
CER8G	Cerebellum_8_L	0.10
**Right amygdala–frontal (49 voxels)**		
F2OD	Frontal_Mid_Orb_R	0.33
FMOD	Frontal_Med_Orb_R	0.15
F1OD	Frontal_Sup_Orb_R	0.12
F3OD	Frontal_Inf_Orb_R	0.12
**Posterior thalamus–hippocampus (39 voxels)**		
HIPPOD	Hippocampus_R	0.33
PARA_HIPPOD	ParaHippocampal_R	0.28
FUSID	Fusiform_R	0.17
CER3D	Cerebellum_3_R	0.11
**Posterior thalamus–visual/lingual (100 voxels)**		
V1G	Calcarine_L	0.47
LINGG	Lingual_L	0.34

Significant group differences were further evaluated at the cluster level using *post hoc* ANOVA (Figure 2B). Significant group effects were maintained at the whole-cluster level [ANOVA; *F*_Group_ (2,62) *p* < 0.001] for: right caudate–cerebellum *F* = 14.96, $\eta_{\text{p}}^{2}=0.35$, right caudate–occipital/fusiform *F* = 15.20, $\eta_{\text{p}}^{2}=0.36$; left caudate–­cerebellum *F* = 14.16, $\eta_{\text{p}}^{2}=0.34$; left amygdala-orbital frontal *F* = 14.16, $\eta_{\text{p}}^{2}=0.34$; posterior thalamus–hippocampus *F*_Group_ = 16.02, $\eta_{\text{p}}^{2}=0.37$; posterior thalamus–occipital/lingual *F*_Group_ = 15.84, $\eta_{\text{p}}^{2}=0.37$; left anterior insula–cerebellum *F*_Group_ = 14.96, $\eta_{\text{p}}^{2}=0.35$. Subsequent group comparisons revealed both marijuana-specific and drug-common effects.

### Marijuana-Specific Effects

+MJ-specific effects comprised four of the seven significant clusters, which are depicted in the uppermost four bar graphs of Figure 2B. +MJ neonates were significantly hypo-connective relative to the −MJ and CTR groups, while −MJ and CTR were statistically indistinguishable (*p* > 0.05) for connectivity between: right caudate–cerebellum (+MJ vs. −MJ/CTR *p* < 0.001); right caudate–occipital/fusiform (+MJ vs. −MJ *p* = 0.003, +MJ vs. CTR *p* < 0.001); left caudate–cerebellum (+MJ vs. −MJ/CTR *p* < 0.001); left anterior insula–cerebellum (+MJ vs. −MJ *p* < 0.001, +MJ vs. CTR *p* = 0.003). *Post hoc* analyses with all categorical drug-exposure effects in the model revealed significant (corrected) marijuana-specific effects for each of these clusters (Table S2 in Supplementary Material). The Opiate drug category was the only non-marijuana drug type to reach marginal significance: left anterior insula–cerebellum (*p* < 0.05 uncorrected).

### Drug-Common and Other Drug Effects

The left amygdala–orbital frontal and posterior thalamus–hippocampus clusters showed a drug-common effect, where both drug-exposed groups differed significantly (corrected) relative to the CTR group and were indistinguishable from each other (+MJ vs. −MJ *p* > 0.05). The left amygdala seed was hyper-connective with orbital frontal cortex (±MJ vs. CTR *p* < 0.001), showing significantly greater positive functional connectivity compared with CTR. The posterior thalamus seed was hypo-connective with hippocampus (+MJ vs. CTR *p* < 0.000, −MJ vs. CTR *p* = 0.002), showing significantly less positive functional connectivity compared with CTR. The posterior thalamus-occipital/lingual was the only cluster demonstrating a specific effect (hypo-connectivity) for the −MJ exposure group (+MJ vs. −MJ *p* < 0.001, −MJ vs. CTR *p* < 0.001, +MJ vs. CTR *p* = 0.573).

### Non-Drug Group Effects

Significant main effects for scanner and gender were detected in two clusters [ANOVA *F*(1,62); *p* < 0.05 corrected]: left caudate–cerebellum, *F*_scanner_ = 8.66 *p* = 0.005, $\eta_{\text{p}}^{2}=0.14$ (Allegra > Trio); posterior thalamus–occipital/lingual, *F*_gender_ = 14.47 *p* < 0.001, $\eta_{\text{p}}^{2}=0.21$ (females > males). Group × non-group interactions for these effects were minimal (see Figure S3 in Supplementary Material). Marginal main effects for gender and birth weight were also detected for two clusters (*p* ≤ 0.05 uncorrected): left amygdala-orbital frontal, *F*_Gender_ = 4.02 *p* = 0.050, $\eta_{\text{p}}^{2}=0.07$; posterior thalamus–occipital/lingual, *F*_birth weight_ = 5.18 *p* = 0.027, $\eta_{\text{p}}^{2}=0.09$, however, these effects were non-significant after correcting for multiple comparisons.

The effects of maternal education and depression, together with other control variables, were tested in a subsample with complete data for these variables (*N* = 46). Main effects and pair-wise group differences were found to be largely consistent with those reported for the reduced model (see Table S3 in Supplementary Material). However, with this smaller sample, group main effects for the left amygdala–PFC and posterior thalamus–hypothalamus clusters did not survive correction but reached marginal significance at *p* = 0.016 and *p* = 0.013, respectively. In this expanded model, maternal education was the only caregiver trait to reach statistical significance [left amygdala–orbital frontal cluster *F*_EDU_(1,45) = 6.05, *p* = 0.019, $\eta_{\text{p}}^{2}=0.14$]. A linear regression analysis revealed a significant negative relationship between functional connectivity and maternal education (see Figure S4 in Supplementary Material); *Z* = −0.0653 (EDU) + 0.39, *r* = −0.47 *p* < 0.001 (connectivity decreased with greater maternal education).

### Effects of Global Signal Regression

For each of these seven clusters, group differences were re-evaluated using data without GSR (i.e., only regressing out motion parameters) and, similarly, motion regressed data with *post hoc* standardization (mean connectivity subtraction). Group-differences and trends were highly consistent with those reported using data with GSR (Figure S5 in Supplementary Material) although some clusters did not reach statistical significance (three out of seven and four out seven, for the two methods, respectively).

## Discussion

We examined the neural correlates of PEM in the neonatal period, focusing on functional connectivity of seed regions with high *in utero* CB1R expression. We found marijuana-specific reductions in bilateral caudate and left anterior insula functional connectivity with cerebellum, and right caudate functional connectivity with occipital/fusiform regions compared with both drug-free and non-marijuana drug-exposed groups. By contrast, both drug-naïve and non-marijuana drug-exposed infants demonstrated co-activations consistent with normative functional connectivity between caudate and cerebellum reported in healthy adults (Postuma and Dagher, 2006; Di Martino et al., 2008; Barnes et al., 2010). Likewise, the lack of anterior insula–cerebellum connectivity in CTR infants we observed in this study replicates our earlier findings in a separate sample of typical unexposed neonates (Alcauter et al., 2015). To our knowledge, this study is the first to detect unique alterations in the brain’s functional organization in the earliest weeks of life in infants with PEM.

Animal studies demonstrate that prenatal exposure to even small amounts of THC disrupts eC signaling during critical periods of brain development, and that PME alters postnatal locomotor activity, cognitive function, and emotional behavior, as well as enhances sensitivity to psychoactive drug use in adulthood (Spano et al., 2007; Campolongo et al., 2011). CB1Rs comprise the largest class of G-protein-coupled receptors in the brain (Freund et al., 2003), with large proportions located in both basal ganglia and cerebellum (Tsou et al., 1998). CB1R is functional and highly expressed early in fetal brain development, even prior to synapse formation. This expression dynamically changes across time and location (e.g., early fetal expression is present in multiple structures, including caudate-putamen, hippocampus, cerebellum, and cortex, followed by later expression in fetal white matter tracts) (Mato et al., 2003). In addition to guiding developing structure and organization, endogenous cannabinoids are also important neuromodulators of (presynaptic) activity in central neurotransmitter systems essential for normal fetal brain development including GABA (Freund and Hajos, 2003), glutamate (Monory et al., 2015), norepinephrine (Cathel et al., 2014) opioids (Wang et al., 2006), and DA (Sanudo-Pena et al., 1999; Dinieri and Hurd, 2012).

### Marijuana-Specific Effects

#### Bilateral Caudate–Cerebellum/Vermis

The caudate, together with the putamen, comprise the striatum, which is the area where approximately 90% of brain DA is released (Kravitz et al., 2015). Rodent models demonstrate that PME alters striatal gene expression of the DA D2 receptor (D2R) (DiNieri et al., 2011). Taken together with the reportedly high striatal CB1R concentration (Glass et al., 1977; Herkenham et al., 1991; Hermann et al., 2015), D2R–CB1R interactions (Anderson et al., 1996) following repeated THC exposure are likely to disrupt developing motor control circuits and may contribute to the current finding of reduced functional connectivity between caudate and cerebellar regions. Both structures play important roles in integrating and coordinating motor and non-motor activity, language, memory, and learning. The cerebellum, which has widespread functional connectivity, primarily to association cortices (Buckner et al., 2011), is thought to calibrate and coordinate, rather than initiate, movements. eC signaling is necessary for cerebellum-dependent discrete motor learning (Kishimoto and Kano, 2006), and for motor adaptation to changing environmental cues/circumstances (e.g., adjusting responses to a moving target). Structurally, bilateral caudate–cerebellar connectivity has been documented with diffusion tensor imaging (DTI) methods (Leh et al., 2007), however, the striatum also has widespread reciprocal connectivity via numerous polysynaptic cortical-striatal loops that may account for correlated functional activations in the absence of monosynaptic anatomical linkage (Kandel et al., 2012). Notably, reduced caudate–cerebellar functional connectivity is reported in patients with Parkinson’s disease, a disorder characterized by degeneration of DA nigrostriatal neurons, and by tremor and later cognitive deficits (Hacker et al., 2012). Conversely, caudate–cerebellar connectivity is increased in heroin-dependent adults (Wang et al., 2013). Therefore, the PME-linked caudate–cerebellar alterations in connectivity we observed may be related to aberrant eC influence on developing striatal dopaminergic afferents or receptor expression, and may partially explain the tremors and enhanced startle responses reported in infants with PME (Fried and Makin, 1987; Fried and O’Connell, 1987), as well as the impaired visual-motor coordination observed in prenatally exposed adolescents (Willford et al., 2010).

#### Left Anterior Insula–Cerebellum

We identified a distinct pattern of relative hypo-connectivity between the anterior insula and the cerebellum in +MJ infants compared with −MJ and CTR groups. Although well-documented in the adult brain (Cauda et al., 2012), anterior insula–cerebellar connectivity was not detected in an earlier sample of typical, unexposed neonates (Alcauter et al., 2015), and this finding is also demonstrated in the CTR group in the current study (see Figures 1 and 2 CTR). The insula is the earliest cortical structure to develop *in utero*, suggesting an important role in development and early survival (Afif et al., 2007). Notably, fMRI reveals that the left insula is activated in newborn infants in response to milk odor (Arichi et al., 2013), which cues the infant to orient and/or move toward mother’s breast for feeding (Varendi and Porter, 2001). As early as the neonatal period, the insula exhibits widespread connectivity, serves as a hub for developing networks and displays anterior-posterior functional specialization (Gao et al., 2011; Alcauter et al., 2015). In adults, anterior insula functional activation has been related to various aspects of self-control including motor impulsivity and reactive aggression (Smith et al., 2004; Dambacher et al., 2015). The insula is thought to link visceral states to conscious feelings and motivations (Noel et al., 2013), and integrates this interoceptive awareness (Noel et al., 2013; Wiebking et al., 2014) with reward properties and salience estimates to compute the value of immediate vs. delayed gratification responses (Volkow and Baler, 2015). These processes are fundamentally linked to drug abuse and addiction (Volkow and Baler, 2015), which is consistent with the enhanced risk for adolescent/adult drug abuse and addiction in both humans and animals with prenatal cannabinoid exposure (Spano et al., 2007, 2010).

#### Right Caudate–Occipital Fusiform

We also found hypo-connectivity between right caudate and right occipital fusiform in the PME group in contrast −MJ and CTR groups. Fusiform gyrus has been related to recognition of faces, words, and emotional content in facial expressions. Right fusiform activation, specifically, is linked to determining whether a “face-like” image is truly a face (Massachusetts Institute of Technology, 2012), and to discrimination of facial identity within a complex visual presentation (Hermann et al., 2015). Reduced local resting state connectivity within the right fusiform is reported in adults with autism spectrum disorder (Itahashi et al., 2015), a disease characterized by impaired perception of facial social cues. The disrupted caudate–fusiform connectivity we observed may compromise visual-spatial processing or motion perception and increase risk for the maladaptive social-emotional behaviors reported in individuals with PME (Gray et al., 2005; Day et al., 2011). Interestingly, negative correlations between dorsal caudate and fusiform are reported in non-drug using adults (Barnes et al., 2010), suggesting that the negative connectivity we observed between right caudate and fusiform areas may reflect a developmental acceleration for infants with PME. However, exact mechanisms and behavioral implications of this observation warrant further investigation.

### Drug-Common Effects

In addition to PME, prenatal nicotine, alcohol, and opiates are well-known neurodevelopmental teratogens. It is important to note that the majority of infants in both +MJ and −MJ groups were exposed to maternal cigarette-smoking and a large proportion to prenatal alcohol consumption. Moreover, although few subjects were opiate users, opiates were more prevalent in the −MJ group. Therefore, the observed drug-common effects may be partially due to these prenatal exposures alone or in interaction with other drugs and/or PME. However*, post hoc* analyses designed to specifically test the effects of individual drug exposures demonstrated both marijuana-specific and non-specific findings that largely parallel the original group-based separation of clusters into marijuana specific (+MJ vs. −MJ/CTR) and drug-common (± MJ vs. CTR), respectively. Mainly, clusters with +MJ specificity only displayed significant effects attributable to marijuana but not to other drugs; clusters with “drug-common” effects showed little drug-specific effects. Thus, the minor differences in drug-exposure profiles (including opiate use in the −MJ group) appear to have minimal effects individually on the observed differences in functional connectivity. Still, future studies with larger samples and more detailed drug-exposure assessments are needed to further tease apart the potential contributions of different drug types, interactions between drugs, and dose-related effects.

#### Left Amygdala-OFC

Both +MJ and −MJ groups demonstrated altered connectivity of amygdala to medial prefrontal/orbital frontal cortex (increased) and of posterior thalamus to hippocampal/medial temporal regions (decreased) compared with the drug-naïve comparison group. Importantly, the medial prefrontal/OFC cluster associated with the left amygdala seed partially overlapped with a cluster detected in our previously reported study of prenatal drug exposure in the larger sample, which included infants with prenatal cocaine, marijuana, nicotine, alcohol, opiate, and SSRI exposures. This finding also parallels reports of increased functional connectivity between amygdala-OFC in chronic heroin users compared with drug-free adults (Ma et al., 2010). Rodent models demonstrate that PME alters opioid precursors in the amygdala and increases opioid seeking in adulthood (Spano et al., 2007). Similarly, prenatal nicotine increases proliferation of enkephalin-producing cells in the central nucleus of the amygdala as well as increasing intake of nicotine and alcohol when offspring reach adulthood (Chang et al., 2013), and prenatal alcohol alters the moderating influence of DA on GABA in the basolateral amygdala (Diaz et al., 2014). Taken together, these findings suggest that the hyper-connectivity between the amygdala and prefrontal regions we observed may represent a final common signature for prenatal exposure to a number of psychoactive drugs, and implies risk for impaired prefrontal inhibition of amygdala responses (Salzwedel et al., 2015).

#### Posterior Thalamus Seed

The posterior thalamus seed also produced a non-specific drug-common alteration of connectivity with a hippocampal/medial temporal cluster. eC signaling in the CA1 region by both interneuron and pyramidal cells regulates activity and network patterns in the developing hippocampus, and this homeostatic control is disrupted by prenatal exposure to cannabinoids (Bernard et al., 2005). However, rodent models also link prenatal nicotine to increased NMDA and reduced nicotinic acetylcholine receptor expression in the hippocampus. These changes are accompanied by long-lasting impairment in learning and memory (Li et al., 2015), which parallel findings of deficits in verbal memory in human children exposed to prenatal nicotine (Fried, 1995; Fried et al., 1997). Similarly, prenatal alcohol causes persistent reductions in hippocampal CA1 and CA3 neuron number and volume that increases with age in non-human primates (Burke et al., 2015).

### Neuroimaging Studies of PME

Few neuroimaging studies of PME have been reported and are limited to subjects tested in late childhood and adolescence. Notably, greater PEM has been linked to alterations in functional activity in young adults, in brain areas involved in spatial working memory and spatial localization, including parahippocampal and cerebellar (increased) and right medial, lateral, and ventral prefrontal regions (decreased) during a visuospatial working memory task (Smith et al., 2004, 2006). In addition, marijuana use in the absence of prenatal exposure is similarly associated with altered connectivity and CB1R expression. Chronic use in adolescence is related to altered functional connectivity in prefrontal networks involved in inhibitory control and executive function (Filbey and Yezhuvath, 2013) and to enhanced prefrontal–cerebellar connectivity at rest and during an attentional control task (Behan et al., 2014). Marijuana-dependent adults show region-specific CB1R down-regulation that is reversible with abstinence (Hirvonen et al., 2012), with greatest down-regulation reported in the caudate, putamen, hippocampus, and nucleus accumbens (Villares, 2007). PME-linked structural differences include impaired white matter microstructure in left frontal callosal projection fibers in 10-year-olds with polydrug exposure, with greatest deficits in those with both PME and prenatal cocaine (Warner et al., 2006). By contrast, findings of reduced cortical and subcortical gray matter volume reported in 10- to 14-year-olds with PME were no longer significant after adjusting for other drug exposures, age, and gender (Rivkin et al., 2008). When taken together, these diverse findings suggest that marijuana use alters brain structure, function, and connectivity, and that the effects of PME may persist into adulthood. However, no studies, to date, have described the effects of prenatal exposure on brain structure or function in neonates, infants, or young children.

### Strengths and Limitations

A major strength of the current study is that brain imaging was done in the first weeks of life, thereby minimizing the effects of postnatal environment to a greater extent than existing reports in which imaging was done years after birth. Confidence in our PME-related findings is enhanced by the inclusion of two comparison groups – one completely drug-free, the other relatively well-matched for sample characteristics, including prenatal exposure to psychoactive drugs other than marijuana. Another advantage is that infants described in this study were born more recently (between 2008 and 2014) than subjects in the majority of existing reports, and therefore, may more accurately reflect current toxicity levels associated with use in pregnant women. This is relevant because marijuana consumption and perceptions of its safety are increasing with the recent trend of legalization (SAMHSA, 2013). Potency has increased six- to sevenfold since the 1970s (Warner et al., 2014), and THC concentration has increased on average from 3.4% in 1993 to 8.8% in 2008 (Mehmedic et al., 2010). Limitations of the study include the relatively small sample size, and the unavoidable replacement of the MR scanner prior to study completion, which resulted in non-random assignment of infant MRI acquisition on two separate Siemens 3 T scanners. However, we did control for scanner in all analyses. Drug group differences in connectivity patterns in the model showing a significant scanner effect (left caudate–cerebellum) were directionally similar for both scanners (Figure S2A in Supplementary Material), and no scanner-by-group interaction was found (*F*_group_ = 12.01 *p* < 0.001, *F*_scanner_ = 8.89 *p* = 0.004, and *F*_group*scanner_ = 0.95, *p* = 0.400). To address the question of scanner effects more definitively, we explicitly tested each cluster for group-by-scanner interactions and found none present. Finally, GSR was used in this study as a preprocessing standardization technique. The use of GSR is widely debated (Saad et al., 2012) though its application in infant studies is potentially advantageous given it has the ability to reduce confounds associated with physiological parameters (Chang and Glover, 2009), which are normally difficult to monitor in naturally sleeping infants. Nonetheless, we re-evaluated our results without GSR and with *post hoc* standardization and found consistent qualitative group differences/trends. These results suggest that although GSR does shift the distribution of correlation values, it does not appear to alter the relative differences and main conclusions of our findings, which are consistent with several of our previous studies (Gao et al., 2013a,b). However, the negative signs of the related functional connectivity values should be interpreted accordingly given the application of GSR.

### Conclusion

Our finding of altered caudate functional connectivity with cerebellum and occipital fusiform, and of anterior insula with cerebellum in +MJ neonates suggests that PME disrupts initial organization of these functional circuits *in utero*. This early departure from typical network development may contribute to the deficits in motor and visual-spatial activity, integration and coordination (Willford et al., 2010), attention (Goldschmidt et al., 2012), and social-emotional stability (Gray et al., 2005) reported in children and adolescents with PME (Fried and Smith, 2001; Fried et al., 2003). These early differences may impair subsequent development of inhibitory control networks that include striatal, cerebellar, and frontal components (Rubia et al., 2007), and may disrupt networks in which the insula serves as a central hub, processing and integrating external information with visceral, cognitive, and affective states to determine salience and guide behaviors, including drug-seeking behavior (Filbey et al., 2009; Feldstein Ewing et al., 2013; Cauda et al., 2014; Wiebking et al., 2014). However, it is not clear whether the results presented here reflect marijuana-related developmental delays or more permanent alterations of functional organization, since brain structure and connectivity continue to undergo dramatic growth in the first 2 years of life (Goldowitz and Hamre, 1998; Knickmeyer et al., 2008; Lin et al., 2008; Gao et al., 2009, 2015). Future longitudinal study that includes measures of structural and functional connectivity as well as cognitive, behavioral, environmental, and more detailed drug-exposure assessments are needed to determine timing and mechanisms that underlie neurobehavioral impairments, and to identify factors that may increase or ameliorate prenatal damage to postnatal brain development in this at-risk group.

## Conflict of Interest Statement

This research was conducted in the absence of any commercial or financial relationships that could be construed as a potential conflict of interest.
