# Supplementary material for: Functional Connectivity Disruption in Neonates with Prenatal Marijuana Exposure
Source: Front Hum Neurosci. 2015 Nov 4;9:601. doi: 10.3389/fnhum.2015.00601 (PMC4631947; doi:10.3389/fnhum.2015.00601)
Supplement: Supplementary file 1 [file table_1.pdf]

Table S1. Frequency of drug use and number of users (*n*) by trimester in +MJ and –MJ groups

		+MJ				-MJ					
	**Trimester**	***n***	**Mean**	**SE**	**Range**	***n***	**Mean**	**SE**	**Range**	***t***	***p***
**Marijuana**	**1**	20	13.29	5.5	0-112	0					
Joints/week	**2**	20	9.12	5.3	0-104	0					
	**3**	18	5.38	3.5	0-69	0					
	**Postnatal**	16	0.34	0.28	0-4.5	0					
**Nicotine**	**1**	14	10.53	1.44	3-20	15	9.77	2.26	0.02 - 40	-0.13	0.899
Cigarettes/day	**2**	13	6.21	1.38	0.01-17	11	10.12	3.24	1.47 - 40	-0.65	0.521
	**3**	8	4.90	1.45	0.65-12	11	6.04	1.38	0.50 - 16	-0.98	0.336
	**Postnatal**	6	4.7	2.38	0.06-15	7	8.45	2.38	0.12 - 20	-1.19	0.245
**Alcohol**	**1**	9	15.23	9.50	0.08-84	6	6.44	4.04	0.08 - 26	1.00	0.347
Drinks/week	**2**	3	0.41	0.18	0.07-0.7	4	4.62	2.64	0.13 - 12	-1.38	0.187
	**3**	2	0.43	0.07	0.36-0.5	3	5.94	3.39	0.69 - 12	-1.32	0.204
	**Postnatal**	1	0.50			2	2.33	1.92	0.42 – 4	-0.97	0.343
**Opiates ***	**1**	0				4	14.88	4.60	7.0 - 24.5		
Times /	**2**	1	2.0			3	12.83	5.83	7.0 - 24.5		
trimester	**3**	0				2	49.0	42.0	7.0 - 91.0		
	**Postnatal**	0				2	19.0	12.0	7.0 - 31.0		

Postnatal: postnatal period before MRI visit; SE: Standard Error; *n:* number of subjects in a group using the drug during that trimester. *The small number of opiate users in each group prevented valid statistical comparison.
