# Supplementary material for: Functional Connectivity Disruption in Neonates with Prenatal Marijuana Exposure
Source: Front Hum Neurosci. 2015 Nov 4;9:601. doi: 10.3389/fnhum.2015.00601 (PMC4631947; doi:10.3389/fnhum.2015.00601)
Supplement: Supplementary file 2 [file table_2.pdf]

Table S2. *Post hoc* analyses of effects of specific drug categories on significant clusters

Seed Cluster	Main Effect	F	p	η_p^2
Right Caudate Cerebellum	Nicotine	0.00	0.978	0.00
	Alcohol	0.26	0.611	0.01
	Marijuana	**21.10**	**0.000**	**0.36**
	SSRIs	1.99	0.167	0.05
	Opiates	0.13	0.722	0.00
Right Caudate Occipital-Fusiform	Nicotine	0.06	0.805	0.00
	Alcohol	0.29	0.591	0.01
	Marijuana	**11.74**	**0.002**	**0.24**
	SSRIs	0.03	0.869	0.00
	Opiates	0.09	0.769	0.00
Left Caudate Cerebellum	Nicotine	0.49	0.489	0.01
	Alcohol	1.57	0.218	0.04
	Marijuana	**20.00**	**0.000**	**0.35**
	SSRIs	0.76	0.390	0.02
	Opiates	0.00	0.972	0.00
L. Anterior Insula Cerebellum	Nicotine	0.03	0.859	0.00
	Alcohol	0.82	0.371	0.02
	Marijuana	**35.69**	**0.000**	**0.49**
	SSRIs	2.86	0.099	0.07
	Opiates	*6.41*	*0.016*	*0.15*
Left Amygdala PFC	Nicotine	0.04	0.846	0.00
	Alcohol	0.20	0.660	0.01
	Marijuana	0.03	0.860	0.00
	SSRIs	0.32	0.574	0.01
	Opiates	0.34	0.565	0.01
Posterior Thalamus Hypothalamus	Nicotine	0.51	0.481	0.01
	Alcohol	0.21	0.648	0.01
	Marijuana	3.48	0.070	0.09
	SSRIs	0.00	0.979	0.00
	Opiates	0.08	0.774	0.00
Posterior Thalamus Medial Visual	Nicotine	1.77	0.192	0.05
	Alcohol	1.17	0.287	0.03
	Marijuana	**14.88**	**0.000**	**0.29**
	SSRIs	3.36	0.075	0.08
	Opiates	0.77	0.387	0.02
