# Supplementary material for: Functional Connectivity Disruption in Neonates with Prenatal Marijuana Exposure
Source: Front Hum Neurosci. 2015 Nov 4;9:601. doi: 10.3389/fnhum.2015.00601 (PMC4631947; doi:10.3389/fnhum.2015.00601)
Supplement: Supplementary file 3 [file table_3.pdf]

Table S3. Summary of main effects and pair-wise group comparisons for full model including all explanatory variables in subset with data for maternal education and depression (N=46)

**Seed Cluster**	Main Effects	**F**	**p**	**η_p^2**	**Group (pair-wise, p)**	
Right Caudate Cerebellum	Group	12.67	0.000	0.41	+MJ vs -MJ	0.000
	G. Age	0.05	0.829	0.00	+MJ vs CTR	0.000
	S. Age	0.52	0.477	0.01	CTR vs -MJ	0.964
	Weight	0.13	0.723	0.00		
	Gender	0.00	0.946	0.00		
	Scanner	1.57	0.219	0.04		
	Maternal EDU	0.48	0.491	0.01		
	Maternal DEP	1.84	0.184	0.05		
Right Caudate Occipital-Fusiform	Group	9.50	0.000	0.35	+MJ vs -MJ	0.049
	G. Age	0.05	0.832	0.00	+MJ vs CTR	0.000
	S. Age	0.11	0.737	0.00	CTR vs -MJ	0.037
	Weight	0.04	0.850	0.00		
	Gender	3.68	0.063	0.09		
	Scanner	0.00	0.946	0.00		
	Maternal EDU	0.44	0.509	0.01		
	Maternal DEP	0.03	0.867	0.00		
Left Caudate Cerebellum	Group	12.52	0.000	0.41	+MJ vs -MJ	0.000
	G. Age	0.62	0.436	0.02	+MJ vs CTR	0.000
	S. Age	0.19	0.669	0.01	CTR vs -MJ	0.608
	Weight	0.16	0.688	0.00		
	Gender	0.00	0.951	0.00		
	Scanner	6.33	0.016	0.15		
	Maternal EDU	0.26	0.614	0.01		
	Maternal DEP	0.60	0.445	0.02		
L. Ant. Insula Cerebellum	Group	9.42	0.001	0.34	+MJ vs -MJ	0.000
	G. Age	0.25	0.621	0.01	+MJ vs CTR	0.029
	S. Age	0.82	0.372	0.02	CTR vs -MJ	0.576
	Weight	0.06	0.813	0.00		
	Gender	0.24	0.630	0.01		
	Scanner	1.94	0.173	0.05		
	Maternal EDU	0.00	0.998	0.00		
	Maternal DEP	0.04	0.852	0.00		
Left Amygdala PFC	Group	4.66	0.016	0.21	+MJ vs -MJ	0.537
	G. Age	0.13	0.726	0.00	+MJ vs CTR	0.369
	S. Age	0.40	0.533	0.01	CTR vs -MJ	0.013
	Weight	0.47	0.496	0.01		
	Gender	3.72	0.062	0.09		
	Scanner	0.21	0.646	0.01		
	Maternal EDU	6.05	0.019	0.14		
	Maternal DEP	0.20	0.660	0.01		
Post. Thalamus Hypothalamus	Group	4.87	0.013	0.21	+MJ vs -MJ	0.363
	G. Age	0.57	0.457	0.02	+MJ vs CTR	0.011
	S. Age	0.02	0.893	0.00	CTR vs -MJ	0.112
	Weight	2.77	0.105	0.07		
	Gender	1.92	0.174	0.05		
	Scanner	0.59	0.446	0.02		
	Maternal EDU	0.03	0.869	0.00		
	Maternal DEP	0.05	0.829	0.00		
Post. Thalamus Medial Visual	Group	13.81	0.000	0.43	+MJ vs -MJ	0.001
	G. Age	0.17	0.682	0.00	+MJ vs CTR	0.940
	S. Age	3.03	0.090	0.08	CTR vs -MJ	0.000
	Weight	7.75	0.009	0.18		
	Gender	7.51	0.009	0.17		
	Scanner	2.10	0.156	0.06		
	Maternal EDU	1.03	0.318	0.03		
	Maternal DEP	0.46	0.500	0.01		
