# Supplementary figures and images for: Functional Connectivity Disruption in Neonates with Prenatal Marijuana Exposure
Source: Front Hum Neurosci. 2015 Nov 4;9:601. doi: 10.3389/fnhum.2015.00601 (PMC4631947; doi:10.3389/fnhum.2015.00601)

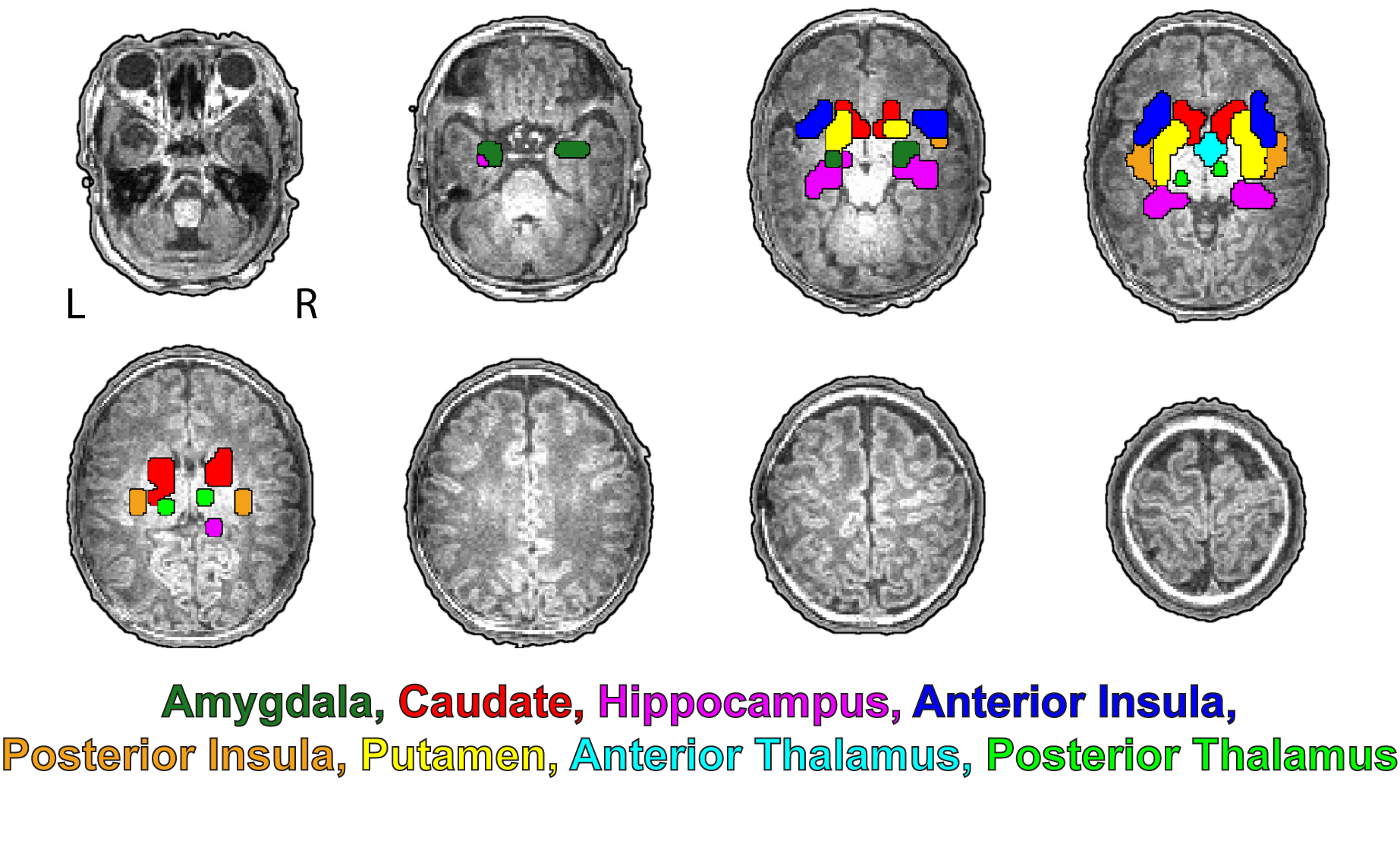

Supplement: Supplementary file 4 [file image_1.tif]

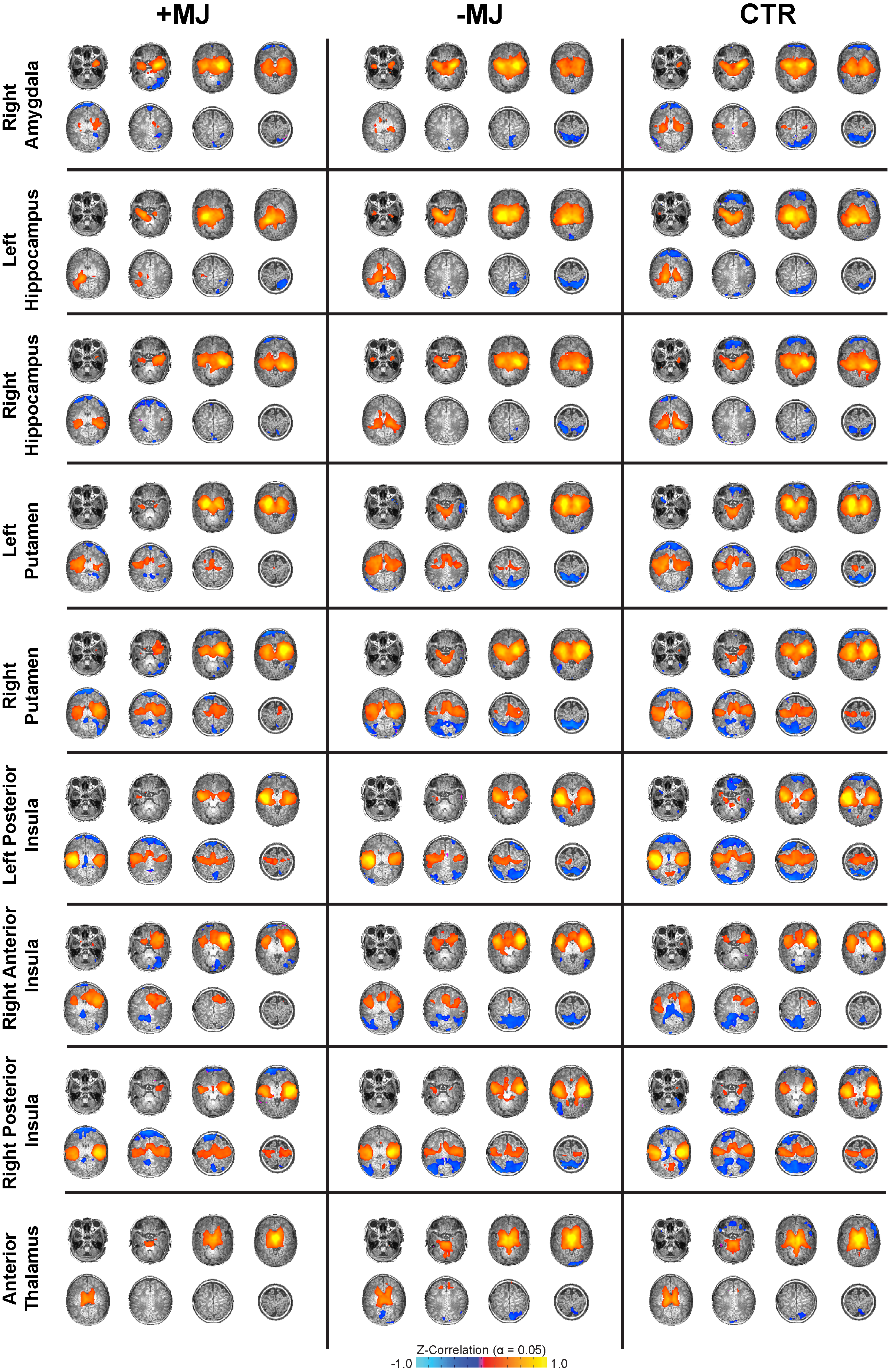

Supplement: Supplementary file 5 [file image_2.tif]

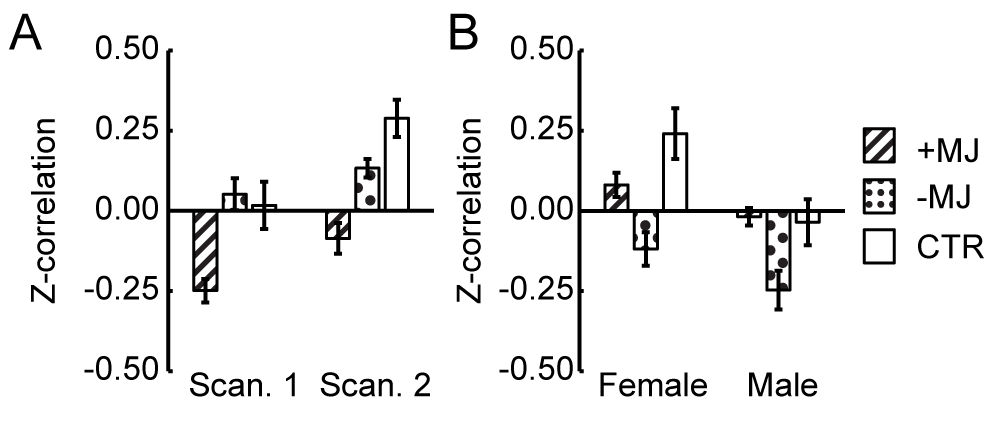

Supplement: Supplementary file 6 [file image_3.tif]

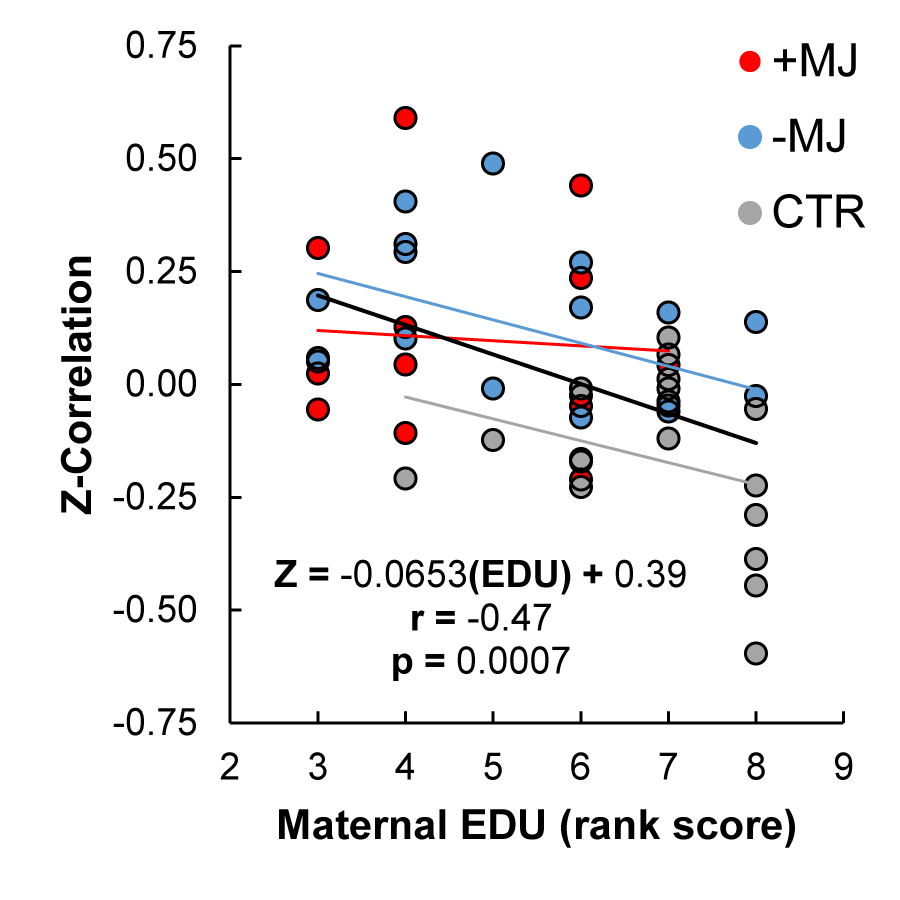

Supplement: Supplementary file 7 [file image_4.tif]

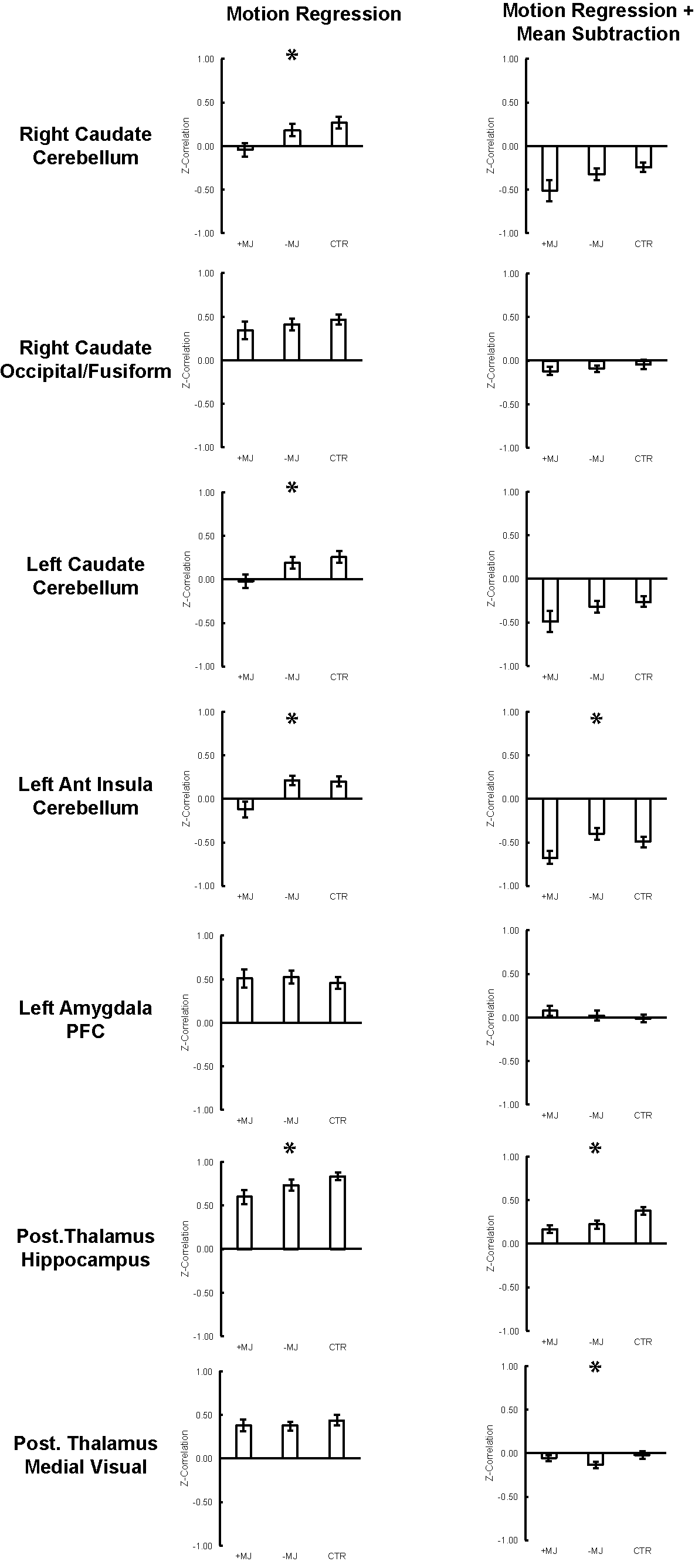

Supplement: Supplementary file 8 [file image_5.tif]
